# Case report: Late middle-aged features of *FAM111A* variant, Kenny–Caffey syndrome type 2-suggestive symptoms during a long follow-up

**DOI:** 10.3389/fendo.2022.1073173

**Published:** 2023-01-04

**Authors:** Yuka Ohmachi, Shin Urai, Hironori Bando, Jun Yokoi, Masaaki Yamamoto, Keitaro Kanie, Yuma Motomura, Yasutaka Tsujimoto, Yuriko Sasaki, Yuka Oi, Naoki Yamamoto, Masaki Suzuki, Hiroki Shichi, Genzo Iguchi, Natsumi Uehara, Hidenori Fukuoka, Wataru Ogawa

**Affiliations:** ^1^ Division of Diabetes and Endocrinology, Department of Internal Medicine, Kobe University Graduate School of Medicine, Kobe, Japan; ^2^ Division of Diabetes and Endocrinology, Department of Internal Medicine, Kobe University Hospital, Kobe, Japan; ^3^ Division of Medical Informatics and Bioinformatics, Kobe University Hospital, Kobe, Japan; ^4^ Clinical and Translational Research Center, Kobe University Hospital, Kobe, Japan; ^5^ Department of Otolaryngology-Head and Neck Surgery, Kobe University Graduate School of Medicine, Kobe, Japan; ^6^ Medical Center for Student Health, Kobe University, Kobe, Japan; ^7^ Division of Biosignal Pathophysiology, Kobe University, Kobe, Japan

**Keywords:** *FAM111A*, hypoparathyroidism, Kenny-Caffey syndrome type 2, short stature, sensorineural hearing loss

## Abstract

Kenny–Caffey syndrome type 2 (KCS2) is an extremely rare skeletal disorder involving hypoparathyroidism and short stature. It has an autosomal dominant pattern of inheritance and is caused by variants in the FAM111 trypsin-like peptidase A (*FAM111A*) gene. This disease is often difficult to diagnose due to a wide range of more common diseases manifesting hypoparathyroidism and short stature. Herein, we present the case of a 56-year-old female patient with idiopathic hypoparathyroidism and a short stature. The patient was treated for these conditions during childhood. Upon re-evaluating the etiology of KCS2, we suspected that the patient had the disorder because of clinical manifestations, such as cortical thickening and medullary stenosis of the bones, and lack of intellectual abnormalities. Genetic testing identified a heterozygous missense variant in the *FAM111A* gene (p.R569H). Interestingly, the patient also had bilateral sensorineural hearing loss and vestibular dysfunction, which have been rarely described in previous reports of pediatric cases. In KCS2, inner ear dysfunction due to Eustachian tube dysfunction may progress in middle age or later. However, this disease is now being reported in younger patients. Nevertheless, our case may be instructive of how such cases emerge chronically after middle age. Herein, we also provide a literature review of KCS2.

## 1 Introduction

Hypoparathyroidism is characterized by decreased parathyroid hormone (PTH) secretion due to impaired PTH activity, presenting hypocalcemia and hyperphosphatemia. The severe symptoms of hypocalcemia include numbness and dysesthesia around the mouth and limbs, tetany, and generalized convulsions (1). Secondary hyperparathyroidism accounts for 75% of all hypoparathyroidism cases. It is attributed to neck surgery, radiation, and various other known causes (2). In patients with congenital hypoparathyroidism, molecular analyses have identified a growing number of causative genes that regulate the formation of parathyroid glands or the synthesis or secretion of parathyroid hormones (3).

In hypoparathyroidism, several factors may account for short stature. These include known endocrine disorders, such as growth hormone deficiency, undernutrition, maternal deprivation syndrome, osteochondral disease, and idiopathic short stature (4). Further, over 1,000 inherited/genetic disorders present with growth retardation as a key feature. For example, Prader–Willi, Turner, and Noonan syndromes often cause short stature (4). Therefore, identifying the genetic causes of short stature is useful for future research. In particular, this may explain the phenotype of idiopathic short stature.

Although hypoparathyroidism and a short stature are caused by many conditions, reports of their coexistence, as in lysine methyltransferase 2D (KMT2D)-related disorders (5) and guanine nucleotide-binding protein subunit alpha 11 (*GNA11*) variants (6), are extremely rare. Thus, diagnosing these diseases presents a challenge. Kenny–Caffey syndrome (KCS) is a rare hereditary skeletal disorder involving hypoparathyroidism and short stature (7, 8).

Based on clinical characteristics and inheritance pattern, KCS is divided into two types: KCS type 1 (KCS1), marked by cognitive development delays, and KCS type 2 (KCS2), marked by average intellect (9). Whole-exome sequencing analyses of *de novo* patients with KCS2 independently identified an FAM111 trypsin-like peptidase A (*FAM111A*) variant, R569H, as a hotspot (10, 11).

Herein, we report the case of a middle-aged female patient with a hotspot variant in *FAM111A* and provide the corresponding literature review. Few reports have been made about the phenotype of KCS2 in older adults. This case presentation may provide an insight into the long-term follow-up of this rare disease during adulthood.

## 2 Case presentation

The patient was a Japanese female born to non-consanguineous parents by normal delivery at approximately 40 weeks of gestation. At birth, the patient weighed 3,000 g and measured 47.0 cm in length. The time when her anterior fontanelle closed is unclear. At six months of age, short stature was noted. At five years of age, cavities in seven teeth were noted.

The patient was referred to our hospital for investigation of short stature. At 11 years of age, the patient measured 115.0 cm (−4.3 SD) and weighed 22.5 kg (−2.8 SD) (**Supplementary Figure**). The patient had low serum Ca and intact PTH levels and was diagnosed with idiopathic primary hypoparathyroidism. Blood relatives had no obvious symptoms of small stature or hypocalcemia. At that time, the disease concept and causative gene of congenital hypoparathyroidism were not yet fully documented; therefore, the patient was treated for hypoparathyroidism with a short stature. The patient was treated with 0.75 µg alfacalcidol OD to maintain serum-corrected Ca levels between 7.2 and 9.2 mg/dL and prevent tetany. The patient presented with various symptoms, which were also treated. The patient was diagnosed with hyperuricemia at age 24 years, gout at age 33 years, and hearing loss at approximately age 49 years. Although the cause of hyperuricemia and gout was unclear, the patient had been treated with benzbromarone for hyperuricemia. There were no obvious tophi in the subcutaneous tissues or joints, and no gout flares over the past decade. The patient also underwent surgery at age 50 years for lumbar spondylolisthesis at L4.

Menarche occurred at age 11 years, and menopause occurred at age 50 years. The Tanner stage of pubic hair and breasts was III. She had never been pregnant and had never given birth.

**Figure 1:**
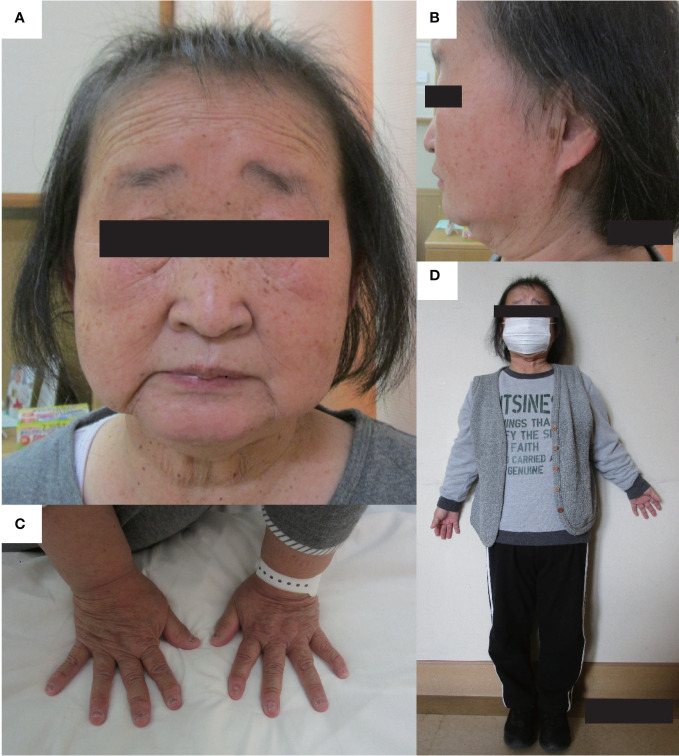
Physical features. The physical features of the patient are shown in panels **(A–D)**. **(A, B)** There are no facial abnormalities, such as cleft palate, low-set auricles, or small mouth; however, the nasal root is flat. **(C)** brachydactyly was shown. **(D)** The patient’s height and arm span were 126.2 cm, and 111.0 cm. Mild disproportionate shortening and short limbs were shown.

**Figure 2:**
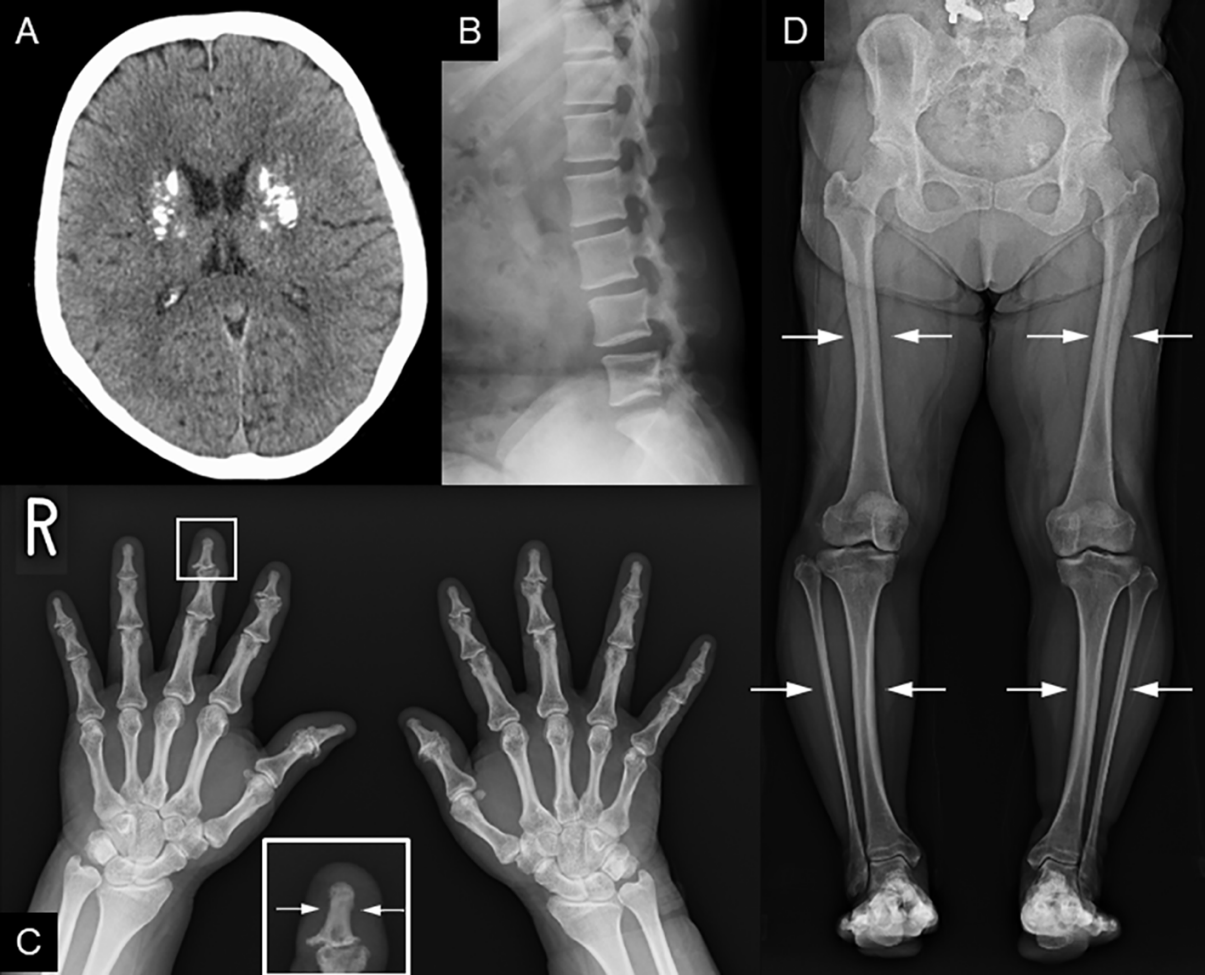
Radiological findings. **(A)** Head CT shows coarse calcification of the capsule. **(B)** Preoperative image of lumbar spondylolisthesis at age 48. Disc space irregularity is seen between L4 and L5. **(C, D)** Cortical thickening of the bones, particularly of the **(C)** fingers and lower leg bones (i.e, femur, tibia, and fibula). **(D)** Medullary stenosis of bones is also observed.

Recently, various etiologies of hypoparathyroidism and short stature have been identified. As such, we reevaluated the patient’s condition. At age 56 years, the patient’s height, arm span, and weight were 126.2 cm (−10.4 SD), 111.0 cm, and 39.3 kg (body mass index: 24.7 kg/m^2^), respectively. Laboratory data showed that the serum-corrected Ca, P, Mg, intact PTH, and 25(OH) Vitamin D levels were 9.1 mg/dL, 4.5 mmol/L, 2.0 mmol/L, 30.0 pg/mL, and 17.6 ng/mL respectively. Thus, hypomagnesemia was less likely to occur. The serum uric acid level was 4.9 mg/dL within normal range. There were no facial abnormalities, such as cleft palate, low-set auricles, or small mouth, which are characteristic of 22q11.2 deletion syndrome (12); however, the nasal root was flat (13). No obvious prominent forehead was observed (**Figure 1**). There was no auricular hypotony or congenital heart disease, which is also a feature of the syndrome. Furthermore, there were no renal abnormalities, which are common features of hypoparathyroidism-sensorineural deafness-renal disease (HDR) syndrome (14). There was no apparent intellectual disability. The patient’s educational attainment was high school level. Head computed tomography (CT) showed coarse calcification of the capsule, which is thought to be a manifestation of chronic hypoparathyroidism (**Figure 2A**). Radiography showed disc space irregularity between L4 and L5 (**Figure 2B**). A skeletal survey showed cortical thickening and medullary stenosis of the bones (**Figures 2C, D**). The patient had no history of fractures and had never been diagnosed with osteoporosis. Dual-energy X-ray absorptiometry showed that the bone mineral density (BMD) T-score reached as high as +1.5 SD in the femoral neck because of abnormal cortical bone thickness. The BMD of the lumbar spine was not available because the scan was performed during the postoperative period.

Notably, the patient had chronic otitis media since childhood. At approximately age 40 years, the patient experienced tinnitus and episodes of dizziness. The patient had mild bilateral sensorineural hearing loss that gradually progressed, especially in the high-frequency range. There was no representative history suggesting the causes of bilateral sensorineural hearing loss, such as the use of ototoxic medications (e.g., aminoglycoside), *in utero* infection, and autoimmune diseases (15). None of her blood relatives had hearing loss. CT showed no significant malformation in the middle and inner ear. An infrared charge-coupled device camera did not show nystagmus. Vestibular function tests were also performed, including the static stabilometer, vestibular-evoked myogenic potential (VEMP), and video head impulse tests (vHIT). In the static stabilometer test, the patient fell during rubber loading and eye closure, suggesting vestibular dysfunction. The patient was unresponsive to bilateral ocular VEMPs, and the vHIT showed reduced vestibulo-ocular reflex gains and catch-up saccades, suggesting bilateral vestibular dysfunction. There were no significant abnormalities characteristic of 22q11.2 deletion syndrome or other conditions. However, a minor dysplasia may have contributed to the recurrent otitis media.

The patient was suspected for KCS2 based on the clinical manifestations of primary hypoparathyroidism, proportionate short stature, cortical thickening, and medullary stenosis of the bones, along with normal intelligence.

The patients have given written informed consent for the use of clinical information and pictures in this report.

## 3 Genetic testing

We performed genetic testing with the approval of the ethics committee of Kobe University Graduate School of Medicine (Approval No. 1646). The patient provided written informed consent for the analysis. Genomic DNA was extracted from whole blood. The Gentra Puregene Blood Kit (QIAGEN, Hilden, Germany) was used according to the manufacturer’s protocol. The purity and quantity of genomic DNA were assessed using a NanoDrop spectrophotometer (Thermo Fisher Scientific, Waltham, MA, USA). The *FAM111A* coding region was amplified from genomic DNA *via* polymerase chain reaction using primers, designed as described previously (10). The *FAM111A* variants were analyzed by Sanger sequencing using the forward and reverse primers described in the previous study, as mentioned above.

**Figure 3:**
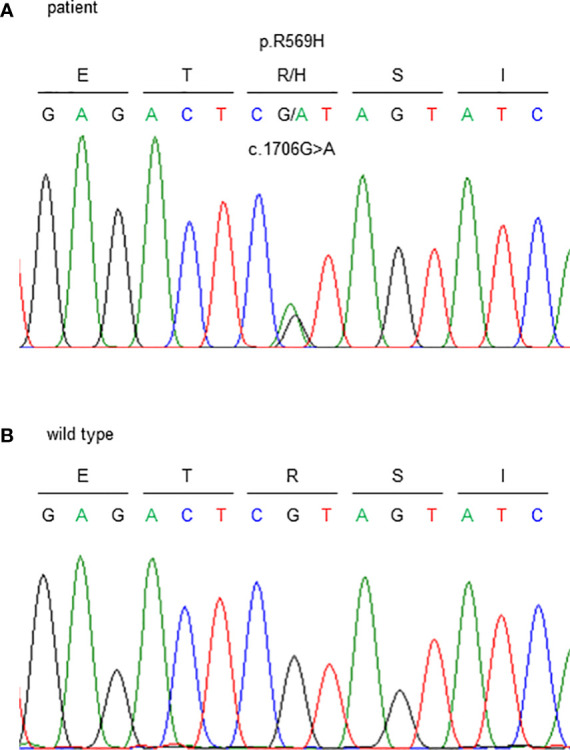
Sanger sequencing of the *FAM111A* gene. c.1706G>A (p.R569H) variants were detected. **(A)** This patient and **(B)** wild type (healthy subject).

Genetic testing identified a heterozygous missense variant in exon 5 of the *FAM111A* gene (NM_001142519.3:c.1706G>A). This resulted in an amino acid substitution of histidine for arginine at codon 569, where the hotspot variant [NP_001135991.1:p.(Arg569His)] causing KCS2 was identified (**Figure 3**). As previously reported (16), this variant was interpreted to be pathogenic, according to the consensus recommendation of the American College of Medical Genetics (17). Thus, the patient was finally diagnosed with KCS2 at age 56 years.

## 4 Literature review

We searched for reports about KCS2 and *FAM111A* variants in PubMed and MEDLINE. The search keywords included “Kenny−Caffey syndrome type 2” or “*FAM111A*”. The available data on clinical evaluations and genetic findings were extracted and summarized (**Table 1**) (10, 11, 16, 18–30).

**Table 1:** Summary of clinical and radiological findings in patients with KCS type 2.

Patient	Author	Year	Origin	*FAM111A* variant (predicted substitution)	Sex	Age at follow-up	Length or stature	Facial features	Eye problems	Skeletal features and imaging	Reference number
**1**	Unger, et al.	2013	Switzerland	c.1706G>A (p.Arg569His)	F	40 years old	−6 SD	Frontal bossing, triangular face, small eyes	Hypermetropia, Cataract	Basal craniosynostosis, V-shaped orbital roof	(11)
**2**	Unger, et al.	2013	India	c.1706G>A (p.Arg569His)	M	17 years old	−6 SD	NA	Hypermetropia	Cortical thickening and medullary stenosis of tubular bones	(11)
**3**	Unger, et al.	2013	Germany	c.1706G>A (p.Arg569His)	M	10 years old	−7 SD	NA	Hypermetropia	NA	(11)
**4**	Unger, et al.	2013	Italy	c.1706G>A (p.Arg569His)	F	Birth, 6 months old	−2 SD −3 SD	NA	not affected	NA	(11)
**5**	Unger, et al.	2013	India	c.1531T>C (p.Tyr511His)	M	7 years old	−5 SD	NA	not affected	Open anterior fontanelle	(11)
**6**	Isojima, et al.	2014	Japan	c.1706G>A (p.Arg569His)	F	2 years old	−4.2 SD	Prominent forehead, deep-set eyes, external ears abnormalities, depressed nasal bridge and micrognathia	Hypermetropia, pseudopapilledema	Cortical thickening and medullary stenosis of tubular bones	(10)
**7**	Isojima, et al.	2014	Japan	c.1706G>A (p.Arg569His)	M	4 years old	−8.2 SD	Prominent forehead, deep-set eyes, depressed nasal bridge and beaked nose, thin upper lip, micrognathia, anteverted nares	Hypermetropia	Cortical thickening and medullary stenosis of tubular bones	(10)
**8**	Isojima, et al.	2014	Japan	c.1706G>A (p.Arg569His)	F	5 years old	−4.5 SD	Prominent forehead, deep-set eyes, beaked nose, thin upper lip, micrognathia	Hypermetropia	Cortical thickening and medullary stenosis of tubular bones	(10)
**9**	Isojima, et al.	2014	Japan	c.1706G>A (p.Arg569His)	M	12 years old	−5.3 SD	Prominent forehead, deep-set eyes, beaked nose, thin upper lip, micrognathia	Hypermetropia	Cortical thickening and medullary stenosis of tubular bones	(10)
**10**	Guo, et al.	2014	Not indicated	c.1706G>A (p.Arg569His)	F	12 years old	−5.99 SD	Prominent forehead, flat nasal bridge, curved and upturned nose	Severe myopia	Bilateral coxa valga, mild medullary stenosis, cortical thickening of long bones	(18)
**11** **(son)**	Nikkel, et al.	2014	Canada	c.1706G>A (p.Arg569His)	F	3 years old	−5 SD	Frontal bossing, small eyes	Wearing glasses	Large anterior fontanelle, medullary narrowing of tubular bones	(19)
**12** **(mother)**	Nikkel, et al.	2014	Canada	c.1706G>A (p.Arg569His)	F	25 years old	−5.5 SD	NA	Wearing glasses	Osteosclerotic skull, cortical thickening and medullary stenosis of tubular bones	(19)
**13**	Kim, et al.	2015	Not indicated	NA (p.Cys485Phe)	M	14 days old	NA	Relative macrocephaly, frontal bossing, large fontanelle, deep-set eyes, a beaked nose	NA	Slender long bones, sclerotic areas in the metaphyseal regions	(20)
**14**	Abraham, et al.	2017	Italy	c.1622C>A (p.Ser541Tyr)	F	6 years old	−3.9 SD	Elfin face with midface hypoplasia, small palpebral fissures, small pinched upturned nose, small chin	not affected	Large anterior fontanelle, multiple wormian bones, overtubulated long bones, metacarpal and metatarsal	(21)
**15**	Wang, et al.	2019	China	c.1706G>A (p.Arg569His)	NA	10 years old	NA	NA	Eye abnormalities	Delayed anterior fontanelle closure, cortical thickening and medullary stenosis of tubular bones	(22)
**16** **(twin 1)**	Cheng, et al.	2020	China	c.1621T>C (p.Ser541Pro)	M	23 years old	−5 SD	Triangular face with mid-face hypoplasia, tall forehead, small palpebral fissures, micrognathia, low set ears, sparse hair	Hypermetropia, astigmatism	Delayed anterior fontanelle closure, cortical thickening and medullary stenosis of tubular bones, prominent odontoid process, tapering fingers with prominent interphalangeal joints	(23)
**17** **(twin 2)**	Cheng, et al.	2020	China	c.1621T>C (p.Ser541Pro)	M	23 years old	−5 SD	Triangular face with mid-face hypoplasia, tall forehead, small palpebral fissures, micrognathia, low set ears, sparse hair	Hypermetropia, astigmatism	Delayed anterior fontanelle closure, cortical thickening and medullary stenosis of tubular bones, prominent odontoid process, tapering fingers with prominent interphalangeal joints	(23)
18*	Cavole, et al.	2020	Brazil	c.1706G>A (p.Arg569His)	M	18 years old	NA	Prominent forehead, narrow nasal base, and broad cheeks	Hypermetropia	Small hands and feet, knee valgus, ankle valgus	(24)
**19**	Turner, et al.	2020	Unknown	c.968G>A (p.G323E)	M	5 months	<2nd percentile	brachycephalic with a large anterior fontanelle, deep set eyes, frontal bossing	not affected	Bicoronal craniosynostosis	(25)
**20**	Deconte, et al.	2020	Unknown	c.1706G>A (p.Arg569His)	F	10 years old	−6.38 SD	Thin nose, micrognathia	Maculopathy (visual impairment)	Short metacarpal bones	(26)
**21**	Kaleta, et al.	2020	Unknown	c.1706G>A (p.Arg569His)	M	12 years old	<3rd percentile	Deep-set eyes, narrowed palpebral fissures, prominent nose, low set ears, prominent frontal bossing	Hypermetropia	Long bones with reduced medullary space, cortical thickening	(27)
**22**	Réka, at al	2021	Not indicated	c.1685A>C (p.Tyr562Ser/ Y562S)	F	20 weeks of gestation	NA	Mild hypertelorism, lowset ears with post-axial rotation and poorly formed helices, and broad and flat nasal root and flat nasal tip	NA	Poorly ossified skull, long extremities, thin diaphyses	(28)
**23**	Yerawar, et al.	2021	India	chr11G>A (p.Arg569His)	F	9 years old	−4.3 SD	Small palpebral fissures, long philtrum, thin upper lip, small pinched nose	Hypermetropia	Thick cortical bone, medullary stenosis of the long bones, absence of diploic space in the skull bones	(29)
**24**	Eren, et al.	2021	Turkey	c.976T>A (c. 1714_1716del)	M	2 months old	−2.73 SD	A relatively large head, small eyes, and inappropriate body size	NA	Narrowing, long, thin bones, thin ribs	(30)
**25**	Lang, et al.	2021	Ireland, Thailand	c.1706G>A (p.Arg569His)	F	7 years old	−2.6 SD	Mild midfacial hypoplasia and retrognathia	Hyper- and hypopigmented macular lesions Nanophthalmos	Mild cortical thickening and medullary stenosis	(16)
**26**	**Ohmachi and Urai, et al.**	**2022**	**Japan**	**c.1706G>A** **(p.Arg569His)**	**F**	**11 years old,** **56 years old**	**−4.3 SD,** **−10.4 SD**	**Flat nasal root**	**not affected**	**Brachydactyly, mild disproportionate shortening and short limbs** **Cortical thickening and** **medullary stenosis of** **tubular bones**	**This study**

NA, not available.

18*: Overlapping phenotypes of Kenny–Caffey type 2 and Sanjad–Sakati syndromes.

## 5 Discussion

In this report, we described the case of a previously reported *FAM111A* variant. Only 26 cases of KCS2, including the current case, have been reported (**Table 1**), suggesting that the hereditary disorder is extremely rare. KCS2 is a relatively new disease. Likewise, *FAM111A* variants have only been identified recently. In fact, we identified a case that was diagnosed only after 45 years. Currently, the majority of the reports are of pediatric cases, with few detailed reports of the disease in adult patients. The number of newly diagnosed cases in adults, such as the present patient, and even children is expected to increase in the future. In one case, hypoparathyroidism was noted in a 2-month-old infant; however, genetic testing was not performed until adulthood (23). Presumably, many cases remain undiagnosed in adults, potentially understating the prevalence of the disease.

Kenny and Linarelli (1966) were the first to describe KCS as an extremely rare genetic disorder (7). Later, Caffey (1967) reported the radiologic findings to correspond to the disease (8). KCS is clinically distinguished by growth retardation, delayed bone maturation, cortical thickening, and medullary stenosis of the long bones, delayed fontanelle closure, ocular and dental abnormalities, hypocalcemia due to hypoparathyroidism, and hypocalcemia-related convulsions (9). In this case, the patient had bilateral sensorineural hearing loss and vestibular dysfunction. Eustachian tube dysfunction due to facial malformations may cause chronic otitis media, causing inner ear damage. Inner ear dysfunction in KCS2 may progress into middle age or later. The hearing and vestibular function of patients with KCS2 have rarely been reported (9, 23); most previous reports are about patients younger than that in the present case.

We have summarized the clinical characteristics of all reported and genetically confirmed cases of KCS2 in **Table 1**. Among the 26 patients, 12 were males (46%), and one was of undetermined sex because the information gathered was insufficient. Currently, no apparent sex predominance has been observed in this disease. Radiography showed that 18 (69%) patients, including our patient, had cortical thickening and medullary stenosis of long tubular bones. Further, 15 (58%), excluding our patient, had refractive anomalies, such as hyperopia. Likewise, although they were visually impaired, none became blind. Finally, except for the present patient, none experienced lumbar spondylolisthesis or hyperuricemia.

According to Moussaid et al., dental issues in KCS2 include failed eruption of permanent dentition, premature loss of teeth, severe dental cavities, oligodontia, and enamel problems (9). At five years of age, our patient had cavities in seven teeth. The link between KCS2 and oral disorders is difficult to establish because many cases of oral disorders are documented immediately after birth. Previous reports suggest the necessity of regular dental visits for patients with KCS2 (9). As KCS2 becomes more widely known, a more detailed profile of this disease is expected in the future because its issues are directly related to the patient's quality of life.

Gout is a common disease caused by a purine metabolic disorder, resulting in uric acid crystal accumulation in the joints and other organs. The onset of gout is frequently associated with a rise in blood uric acid levels (31). Gout is more prevalent in men than in women and is associated with increasing age (32). With better living conditions and changes in nutrition, the incidence of gout has increased and tends to manifest at a younger age. Considering the epidemiology of gout, this case was unusual because it affected a woman at a younger age. Recently, several studies have shown that gene alterations may play a significant role in hyperuricemia and gout development; however, the mechanism or genetic etiology has not been fully confirmed (33). The association of *FAM111A* variants with hyperuricemia remains unclear. Hyperuricemia has been closely related to cardiovascular disease and chronic kidney disease. Accumulation of more adult cases and long-term follow-up studies are needed to investigate the association of the *FAM111A* variant with potential risk factors for hyperuricemia and cardio-metabolic disease.

Sensorineural deafness, lumbar spondylolisthesis, and hyperuricemia were unique symptoms in our case. As discussed above, there have been no definite reports of a relationship between these symptoms and KCS2. It has been reported that other genetic disorders caused by phosphoribosylpyrophosphate synthetase 1 (*PRPS1*) mutations, which causes hyperuricemia due to overproduction of purine, are accompanied by sensorineural hearing loss (34, 35); however, we could not perform whole exome sequencing in the present case to investigate the possibility that KCS2 overlaps with such other genetic disorders. Further case accumulation and investigation are needed to understand whether these were unique to this patient or are related to the disease. If these symptoms were common in middle-aged elderly persons with KCS2, we should pay attention to them during a long follow-up.

According to a 2013 study, the autosomal dominant form of KCS, KCS2 (OMIM 127000), is caused by variants in the gene-encoding family with sequence similarity 111 member A, *FAM111A* (OMIM*615292) (13). In some cases, *de novo* variants occur. The autosomal recessive form of KCS, KCS1 (OMIM 244460), is genetically and clinically distinct from KCS2. KCS1 is associated with homozygous or compound heterozygous variants in the tubulin-specific chaperone E gene (36). Kenny and Linarelli were considered the first to report a case of KCS2 because their case involves an autosomal dominant pattern of inheritance (7). The absence of prenatal growth and intellectual disability distinguishes KCS2 from KCS1 (9).

*FAM111A* encodes a protein comprising 611 amino acids. However, its functions are not completely understood (10). Unger et al. suggested that this gene plays a crucial role in skeletal development, parathyroid hormone synthesis, and calcium and phosphorus homeostasis (13). The phenotypic manifestations of KCS1 and KCS2 show that *FAM111A* pathogenic variants negatively affect postnatal growth, neural development, and bone development. Fine et al. reported that *FAM111A* plays an important role in viral infection. It interacts with simian virus 40 large T antigen, restricting host range function, as well as virus survival and replication (36). Nie et al. suggested that *FAM111A* variants in KCS2 and osteocraniostenosis (OCS) are hyperactive and cytotoxic, inducing apoptosis-like phenotypes, such as disruption of nuclear structure and pore distribution in a protease-dependent manner. In this regard, nucleoporins and germinal-center-associated nuclear protein transcription and replication factors have been identified as *FAM111A* interactors and candidate targets. Ultimately, Nie et al. discovered a potentially unifying mechanism in which dysregulated *FAM111A* activity limits viral replication, resulting in KCS2 and OCS (37). However, how these variants caused the observed phenotypes remains unclear.

The autosomal recessive inheritance pattern of KCS must be considered in the differential diagnosis of idiopathic hypoparathyroidism. Other conditions to consider include 22q11.2 deletion syndrome, which presents with characteristic facial features; HDR syndrome, which is marked by sensorineural hearing loss; and autoimmune polyendocrinopathy syndrome type 1 (4). Therefore, physicians must carefully check for the features of each disease to make the appropriate diagnosis. In addition, genetic testing is essential for a definitive diagnosis.

Questions regarding the clinical features, age-related changes, pathogenesis, and treatment of KCS remain unanswered. Therefore, further accumulation of cases is required in the future, and physicians must be informed about KCS.

## 6 Conclusions

This case report highlighted the clinical, biochemical, and radiological characteristics of an adult patient with KCS2 caused by the *FAM111A* variant p.R569H. This finding is consistent with those of recent independent reports. Although this disease is now being reported in younger patients, our case may be instructive of how the disease presents in aging.

## Data availability statement

The original contributions presented in the study are included in the article/**Supplementary Material** further inquiries can be directed to the corresponding author.

## Ethics statement

The studies involving human participants were reviewed and approved by ethics committee of Kobe University Graduate School of Medicine (Approval No. 1646). The patient provided their written informed consent to participate in this study. Written informed consent was obtained for the publication of this case report.

## Author contributions

YOh, SU, HB, JY, and NU contributed to the writing of the manuscript. YOh, HB, JY, MY, KK, YM, and NU made a clinical diagnosis. SU performed a genetic diagnosis. YT, YS, YOi, NY, MS, HS, and GI searched previously reported cases for the literature review. HF and WO contributed to critical revision of the article for important intellectual content. All authors contributed to the article and approved the submitted version.

## Supplementary material

Supplementary Figure Growth chart. Height and weight plots at each age (38).
