# Supplementary figures and images for: Case report: Late middle-aged features of FAM111A variant, Kenny–Caffey syndrome type 2-suggestive symptoms during a long follow-up
Source: Front Endocrinol (Lausanne). 2023 Jan 4;13:1073173. doi: 10.3389/fendo.2022.1073173 (PMC9846794; doi:10.3389/fendo.2022.1073173)

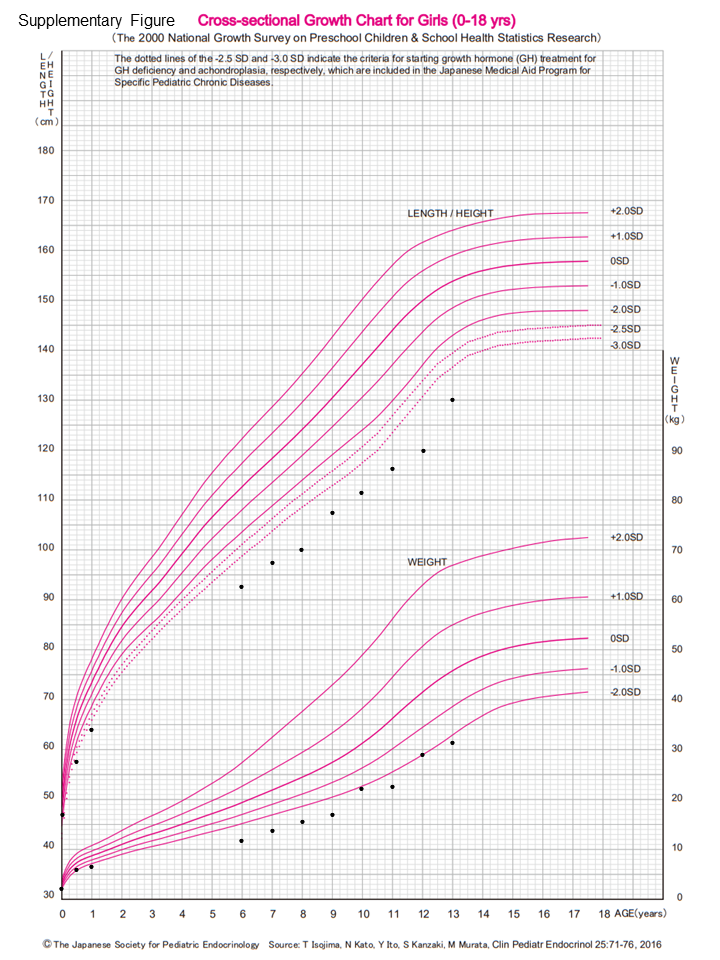

Supplement: Supplementary Figure — Growth chart. Height and weight plots at each age (38). [file Image_1.tif]
